# Smoking intensity and bladder cancer aggressiveness at diagnosis

**DOI:** 10.1371/journal.pone.0194039

**Published:** 2018-03-23

**Authors:** André L. A. Barbosa, Sita H. H. M. Vermeulen, Katja K. Aben, Anne J. Grotenhuis, Alina Vrieling, Lambertus A. Kiemeney

**Affiliations:** 1 Department for Health Evidence, Radboud Institute for Health Sciences, Radboud university medical center, Nijmegen, The Netherlands; 2 Federal University of Bahia, Salvador, Brazil; 3 Netherlands Comprehensive Cancer Organisation, Utrecht, The Netherlands; 4 Department of Urology, Radboud Institute for Health Sciences, Radboud university medical center, Nijmegen, The Netherlands; Centro Nacional de Investigaciones Oncologicas, SPAIN

## Abstract

**Objective:**

To explore the relation between cigarette smoking intensity and bladder cancer aggressiveness at first diagnosis.

**Methods:**

Patients diagnosed with urinary bladder cancer (BC) between 1995–2011 under the age of 75 years were retrospectively identified from the Netherlands Cancer Registry and invited for a study on genetic and lifestyle risk factors for BC. Information on patients’ self-reported smoking history was retrieved by means of a postal questionnaire. Tumors were stratified regarding the risk of progression defined by tumor stage and grade. Multinomial logistic regression was used to analyze the relation between smoking intensity and aggressiveness of the tumor.

**Results:**

The UBC study population comprised 323 (17.4%) never smokers, 870 (46.8%) former cigarette smokers, and 630 (33.9%) current cigarette smokers. A higher smoking amount was a risk factor of getting high-risk non-muscle invasive bladder cancer (NMIBC) compared with low-risk NMIBC in ever and former cigarette smokers (OR: 1.02 per cigarette smoked, 95% CI: 1.00–1.03 and OR: 1.03, 95% CI: 1.01–1.05, respectively). A statistically significant dose-response increase in the risk of a more aggressive cancer type (high-risk NMIBC and MIBC) was observed with increasing smoking duration among former smokers (p for trend 0.035 and 0.008, respectively). No significant association of the evaluated smoking intensity variables was observed in current smokers. A longer time of smoking cessation correlated with a lower odds of a more aggressive cancer.

**Conclusion:**

We observed a weak increase in the risk of a more aggressive tumor type with increasing smoking intensity in former smokers, but this association was absent in current smokers.

This conflicting result may suggest that there is no strong relation between smoking intensity and bladder cancer aggressiveness. Analyses of prospective studies with longitudinal smoking assessment may provide a more definitive answer to the research question.

## Introduction

Worldwide, urinary bladder cancer (BC) is the fifth most prevalent type of cancer among men, while it ranks twelfth among women [1]. In high income countries, >90% of all BC are urothelial cell carcinomas (UCC), the remaining mainly being squamous cell, adeno or small cell carcinomas [2]. BC comprises a heterogeneous group of tumors that arise by different molecular pathways. Relatively benign, low grade papillary tumors are characterized by loss of heterozygosity of chromosome 9 and mutations in *FGFR3*, *PIK3CA* and *STAG2*. Muscle-invasive bladder cancer (MIBC) commonly shows *TP53* mutations and *RB1* defects. Carcinoma *in situ* (CIS) and a small part of the T1 tumors may have the same characteristics as MIBC [2]. Approximately 75% of patients with BC present with a tumor confined to the mucosa or submucosa of the bladder wall, *i*.*e*., non-muscle-invasive bladder cancer (NMIBC), characterized by a relatively good prognosis but high relapse rate. On the other hand, the 25% of patients with MIBC (including metastatic disease) are at considerable risk of cancer-specific mortality [3–5].

Tobacco smoking, occupational exposure to aromatic amines and other carcinogens, genetic factors, pelvic radiation therapy, and cyclosphosphamide chemotherapy are risk factors for UCC [6]. Schistosomiasis infection and chronic inflammation secondary to bacterial infections, indwelling catheters, bladder calculi or chronic bladder outlet obstruction are also risk factors, although more related to squamous cell carcinoma (SCC) [7,8]. Smoking is recognized as the most important risk factor for BC and is estimated to account for 50% of all tumors [9]. Tobacco contains multiple carcinogens, such as 4-aminobiphenyl, 2-naphthylamine, and aromatic amines which are associated with BC induction in smokers [10]. The risk of bladder cancer increases with the number of cigarettes and years smoked [11].

Some studies suggest that a higher intensity of smoking is significantly related to a more aggressive bladder tumor at diagnosis [12–19], while other studies found no association between smoking intensity and grade or stage of the tumor [20–28]. Table 1 summarizes the current evidence on the relation between smoking intensity and tumor characteristics. If a relation between the amount and duration of smoking and tumor aggressiveness exists, it may improve the doctor’s ability to identify patients at risk of a more severe tumor type. Also, it may be one more reason to stimulate counseling for smoking cessation. This study explores the association between the intensity of smoking and the aggressiveness of UBC at first diagnosis in a large population-based BC series from the Netherlands.

**Table 1:** Studies that evaluated the relation between smoking intensity and tumor characteristics.

Article	Study Design	Population	Aggressiveness definition	Smoking intensity definition	Main findings	Comments
**Pietzak et al. 2015 (12)**	Historical cohort study	740 from Pennsylvania (USA)	Tumor grade (high vs. low) and stage (Tis, Ta, T1, T2, T3 e T4). Muscle-invasive disease.	Non-smokers, light-smokers (≤30 pack/years), and heavy-smokers (>30 pack/years). Criteria for National Lung Cancer Screen Trial, age of 55–74 years with ≥30 pack-years smoking exposure and <15 years of smoking cessation	Heavy smokers were also more likely to have an initial tumor, which was high grade with a more advanced clinical stage. On multivariate analysis shown in pack-years smoking exposure did not remain significantly associated with muscle-invasive disease. Pack-year was associated with an increased risk of an initial high-grade tumor	On multivariate analysis, meeting screening criteria was independently associated with an initial muscle invasive tumor and also associated with an increased risk of an initial high-grade.
**Rink et al. 2013 (13)**	Historical cohort study	1506 from North America and Europe	Tumor stage, grade, lymphovascular invasion and lymph node metastasis. ≥pT3 and/or pN+ as advanced tumor stage	Cumulative smoking exposure: Light short-term smokers (≤ 20 cig/day for ≤ 20 y), heavy short-term smokers (>20 cig/day for ≤ 20 y), light long-term smokers (≤20 cig/day for >20 y), and heavy long-term smokers (>20 cig/day for >20 y). Duration of smoking (≤ 10, 11–20, 21–30, or >30 y). Quantity of smoking (1–10, 11–20, 21–30, or >30 cig/day.	When ever smokers were categorized by cumulative smoking exposure, tumor stage (p = 0.007), and lymph node metastasis (p = 0.003) and lymphovascular invasion (p = 0.030) differed by cumulative smoking exposure. In multivariable logistic regression analyses, smoking Duration and cumulative smoking exposure were each significantly associated with advanced tumor stage after adjusting for the effects of age, gender and study center.	
**van Roekel et al. 2013 (20)**	Prospective cohort study	1.067 from West Midlands (UK)	Higher vs. lower T stage, grade and tumor size (mean), NMIBC vs. MIBC, multiplicity vs. solitary	Smoking frequency (g/day) and cumulative smoking amount (Kg).	No associations between smoking intensity measures and tumor characteristics.	A significant dose-response relationship was found between higher smoking frequency and lower age at diagnosis.
**Chamssuddin et al. 2013 (14)**	Historical cohort study	300 from Syria	Grade: low-grade (G1) vs. high-grade (G≥2). Stage: low-stage (Ta + T1) high-stage (T≥2)	Dose: low- (10–29 cig/day), moderate- (30–59 cig/day) and high-dose smokers (≥60 cig/day)	Comparing the high-, moderate- and low-dose smokers, the high-dose group had significantly higher grades and stages than the other groups. The difference between moderate- and low-dose smokers was not significant for grade or stage.	
**Mitra et al. 2013 (21)**	Historical cohort study	212 from Los Angeles	Stage: Non-muscle-invasive (Ta/ T1/CIS, N−), muscle-invasive (T2−4, N−) and nodal metastasis (any T, N+)	Group 1 (nonsmokers + smoker ≤20 cig/day for ≤30 y), group 2 (smoker 31–40 y + smoker >20 cig/day for ≤ 30y) and group 3 (smoker >40 y)	The distribution of the smoking intensity group does not show statistical differences according to tumor stage.	Nonsmokers were combined with light smokers in group 1 as a full sensitivity analysis revealed no substantive outcome differences between the two subgroup
**Ajili et al. 2013 (22)**	Historical cohort study	81 from Tunisia	Multiplicity (single or multiple). Histological grade (low, high). Stage (pTa or pT1). Size (<3 cm or ≥3 cm)	Smoking intensity: ≤ 60 pack-years vs. >60 pack-years	There was no association between smoking intensity and tumor multiplicity, grade, stage and size.	
**Jiang et al. 2012 (15)**	Case-control study	1.439 cases and 1,586 controls from Los Angeles (USA)	Low-grade superficial tumors (Ta and grade<3) vs. high grade superficial tumors (Ta grade 3 and T1) vs. muscle-invasive tumors (T2-T4)^1^.	Daily dose (cig/day) and duration (years of smoking).	Compared to non-smokers, heavy smokers (i.e., >40 cig/day and >40 years of smoking) had higher risk of invasive bladder cancer (OR = 9.0, 95% CI = 4.8–16.8) than for low-grade superficial bladder cancer (OR = 3.6, 95% CI = 2.3–5.8)	Also a higher risk of MIBC was found for higher daily dose and for longer smoking duration.
**Ros et al. 2012 (23)**	Prospective cohort study	468,656 from 10 European countries	Aggressive (≥T1, CIS or G3) vs. non-aggressive (TaG1 or TaG2)	Smoking duration (y) and lifetime number of cigarettes (cig/day)	No association between smoking behavior and aggressiveness of the tumor among men. And not consistent finding in women.	Differences on the distribution of smoking behavior among aggressive or non-aggressive subgroups were not tested.
**Ouerhani et al. 2009 (24)**	Cohort study	80 from North of Tunisia	Superficial (Ta + T1) vs. Invasive ≥T2. Grade (G1 vs. G2). Tumor Groups (T1G2 vs. TaG3 + T1G3 vs. ≥T2)	Nonsmoker, smoker of <40 pack year and smoker of ≥40 pack year.	The comparison of pack year according to tumor stages and grades does not show significant statistical differences.	
**Serretta et al. 2006 (25)**	Prospective cohort study	474 from Italy	Stage (Ta vs. T1). Grade (I vs. II). Number (single vs. multiple).	Period of smoking (>20 y vs. ≤20 y) and cig/d (>20 vs. ≤20)	No statistical correlation was found between period of smoking and cig/day with stage, grade and number.	
**Mohseni et al. 2004 (16)**	Case-control study	185 from Tehran (Iran)	High-grade (3) vs. low-grade (1–2)). Size (small tumor (<2cm) vs. moderate (2-5cm) vs. large (>5cm).	History of smoking (pack-years).	Cigarette smoking rate was statistically higher in patients with high-grade tumors.	Number of cases and controls not specified.
**Persad et al. 1997 (26)**	Case-control study	107 cases and 85 controls from Bristol (England)	Grade as aggressive (GIII) vs. non-aggressive (GI and GII). Stage (Tis, Ta, T1, T2, T3 and T4)	Pack-year (mean)	No evidence that grade and invasiveness were associated with greater exposure.	
**Sturgeon et al. 1994 (17)**	Case-control study	2,982 cases and 5,782 control from Connecticut, Iowa, Utah, New Mexico, metropolitan area of Atlanta, Detroit, San Francisco and Seattle (USA)	Grade (I vs. II vs. III/IV). Stage (noninvasive (*In situ*, confined to mucosa) vs. confined to submucosa vs. muscle invasion vs. extension beyond bladder.)	Cigarette use: never, ex-smoker (<20 cig/day or ≥20 cig/day) and current smoker (<20 cig/day, 20–39 cig/day or ≥40 cig/day)	Risk of each stage of bladder cancer increased with cigarette smoking, but the more advanced the stage, the higher the relative risk. Grade of bladder cancer at diagnosis varied little according to cigarette smoking.	Within both the non-invasive and invasive tumor stratum, cigarette use was more strongly associated with low-grade than high-grade bladder cancer.
**Hayes et al. 1993 (18)**	Case-control study	368 cases and 466 controls from Massachusetts (USA)	Invasive (≥ T1) vs. superficial (Ta and Cis).	<0.5; 0.5–1.4 and 1.5+ packs of cigarettes per day.	The higher amount of packs of cigarettes per day increases the risk of invasive cancer.	For superficial tumors, the risk was elevated for all tobacco-use levels, there was no clear dose-response trend.
**Brooks et al. 1992 (27)**	Historical cohort study	2,893 from Missouri (USA)	Low grade (G1,G2) vs. high (G3,G4)). Early stage (Tis, Ta, T1) vs. late (T2,T3,T4))	Smoking status: never, former and light (< 1 pack per day), moderate (1–2 packs per day) heavy (>2 packs per day)	There is no trend toward higher grade disease as smoking increased from light to moderate to heavy. Late stage cancer: OR = 1.2 (light), OR = 1.6 (moderate) and OR = 1.7 (heavy) (not statistically significant trend).	
**Jensen et al. 1987 (28)**	Case-control study	790 cases and 389 controls from Copenhagen and Frederiksberg (Denmark)	Grade (3–4 vs. 1–2). Stage (T2-T4 vs. Ta-T1).	Pack-years and pack-year equivalents.	No association was found between pack-years and the characteristics of the tumor (stage and grade).	
**Morrison et al. 1982 (19)**	Historical cohort study	762 from Greater Boston, Massachusetts, USA, 583 from part of Greater Manchester County, UK, and 348 from metropolitan Nagoya, Japan.	Grade (0-I vs. II vs. III).	Current smokers: <1 packs/day vs. 1 pack/day vs. 2+ packs/day.	The percentage of grade-III tumors among current smokers in each area increased irregularly with the amount smoked (packs/day).	Cigarette smoking was not consistently related to histologic type

## Materials and methods

### Study population

This study used data from the Nijmegen Bladder Cancer Study (NBCS). This study population has been described in more detail previously [29]. Briefly, BC patients diagnosed between 1995–2011 under the age of 75 years in the mid-eastern part of the Netherlands were identified through the Netherlands Cancer Registry (NCR) held by the Netherlands Comprehensive Cancer Organization (IKNL) and contacted via their treating physicians.

Patients who consented to participate in the study were asked to fill out a lifestyle questionnaire, including questions on education, occupation, medical history, physical activity, and complete history of smoking. Furthermore, blood samples were collected by Thrombosis Service centers, which hold offices in all the communities in the region. The study was approved by the institutional review board of the Radboud university medical center, Nijmegen, The Netherlands (CMO Arnhem-Nijmegen).

### Smoking assessment

Information on smoking history was obtained via the lifestyle questionnaire. Patients were asked for their smoking status at recruitment, age at smoking initiation and cessation, number of cigarettes, pipes and cigars smoked per day and duration of smoking in years. The timing of smoking cessation with respect to the diagnosis was calculated as age at diagnosis minus age at cessation. Smoking status at diagnosis was classified as never smoker, former smoker (quitted >1 year before diagnosis), current smoker (continuing cigarette smoker or quitted ≤ 1 year before diagnosis). The cutoff point of 1 year before diagnosis was chosen for 2 reasons. First, because we believe that most patients with early symptoms will be diagnosed within a year, and second because a change in smoking habits in the year before diagnosis will probably not have any major effect on bladder cancer aggressiveness. Ever smokers were defined as the combination of former and current smokers. In the current smokers group, only the smoking period in years before the diagnosis was considered. Smoking amount was evaluated as cigarettes per day. Cumulative smoking exposure (in pack-years) was calculated by multiplying the cigarette smoking duration and packages per day (20 cigarettes representing one package). Pipe and/or cigar smoking (5.9% of all patients) was ignored in the main analyses, assuming that the majority of Dutch pipe and cigar smokers do not inhale the smoke [30].

### Outcome assessment

Detailed clinical data concerning age at diagnosis, tumor stage, tumor grade, tumor number (single or multiple), tumor size (<3cm and ≥ 3cm), presence of concomitant CIS, and histological type were collected through a medical file survey. Tumor stage and grade were recorded according to the final conclusion in the pathology report. Tumors with WHO 1973 differentiation grade 1 or 2, WHO/ISUP 2004 low grade, or Malmström (Modified Bergkvist) grade 1 or 2a were considered low-grade tumors. We classified tumors with WHO 1973 differentiation grade 3, WHO/ISUP 2004 high grade, or Malmström (Modified Bergkvist) grade 2b or 3 as high-grade [31,32]. Tumor aggressiveness was classified according to the risk of progression as follows: low-risk NMIBC (low-grade Ta tumors), high-risk NMIBC (all stage T1 tumors, all high-grade tumors, or CIS) and MIBC (stage ≥ T2 or any stage with ≥N1 and/or M1) [33].

### Statistical analysis

Patient and tumor characteristics were compared between the smoking status categories using chi-square, Fisher exact, and one-way analysis of variance (ANOVA) tests where appropriate. Because it is generally believed that there is no ‘no effect level’ in the relation between smoking and the risk of cancer, we analyzed the dose of smoking using continuous variables. The distribution of continuous smoking variables was compared between the categories of tumor multiplicity and tumor aggressiveness and tested for statistical significance using the non-parametric Kruskal-Wallis test. Multinomial logistic regression was used to analyze the relation between smoking intensity and aggressiveness of the tumor with adjustment for gender and age at diagnosis. Low-risk NMIBC was considered as the reference group. We repeated similar analyses for tumor multiplicity as the dependent variable using solitary tumors as the reference group. The association of each smoking intensity variable (smoking amount, smoking duration and cumulative smoking exposure), age at smoking initiation, and time since smoking cessation was assessed separately in ever, former and current smokers. Statistical analysis was performed using IBM SPSS Statistics for Windows 20 (IBCM Corp., Armonk, NY, USA) with a p value < 0.05 indicating statistical significance. The data have been made publicly available thru the Data Archiving and Networked Services (DANS-EASY) and can be accessed via the link http://dx.doi.org/10.17026/dans-2a6-ate2.

## Results

A total of 1859 BC patients were included in the study. The majority of the patients were men (81.1%). The mean age at diagnosis (SD) was 62.4 (±9.7). Never cigarette smokers represented 323 (17.4%) of all patients, former cigarette smokers 870 (46.8%), and current cigarette smokers, 630 (33.9%). Of all patients, only 40 had a non-UCC histology. Because their smoking distribution was quite similar (Never 25%, Former 40% and Current 30%) to that of the UCC patients, we decided not to exclude these 40 patients. A comparison of patient and tumor characteristics by smoking status is shown in Table 2. The groups differ from each other in age at diagnosis, gender, history of cigar or pipe smoking and tumor stage.

**Table 2:** Baseline characteristics of study population.

N (%)	Total population (n = 1,859)	Never cigarette smokers (n = 323)	Former cigarette smokers (n = 870)	Current cigarette smokers (n = 630)	Unknown smoking status (n = 36)	*p* value^1^
**Age at diagnosis (mean ± SD)**	62.4 ±9.7	61.5±11.3	65.1±8.3	59.0±9.5	66.9±8.3	<0.001
***Gender***						<0.001
**Male**	1,507 (81.1)	215 (66.6)	757 (87.0)	503 (79.8)	32 (88.9)	
**Female**	352 (18.9)	108 (33.4)	113 (13.0)	127 (20.2)	4 (11.1)	
***Type of tobacco***						<0.001
**Ever cigar and/or pipe smoker**	369 (20.2)	109 (33.7)	170 (19.6)	85 (13.5)	5 (71.4)	
**Never cigar and/or pipe smoker**	1458 (79.8)	214 (66.3)	697 (80.4)	545 (86.5)	2 (28.6)	
**Missing**	32	0	3	0	29	
***Tumor stage***						0.036
**0a**	1,052 (57.4)	187 (59.0)	489 (57.1)	359 (57.7)	17 (47.2)	
**0is**	59 (3.2)	13 (4.1)	27 (3.2)	16 (2.6)	3 (8.3)	
**I**	400 (21.8)	68 (21.5)	208 (24.3)	117 (18.8)	7 (19.4)	
**II/III/IV**	322 (17.6)	49 (15.5)	134 (15.6)	130 (20.9)	9 (25.0)	
**Missing**	26	6	12	8	0	
***Concomitant CIS***						0.341
**No**	1614 (88.1)	284 (89.3)	744 (87.0)	555 (89.2)	31 (86.1)	
**Yes**	217 (11.9)	34 (10.7)	111 (13.0)	67 (10.8)	5 (13.9)	
**Missing**	28	5	15	8	0	
***Tumor grade*^*2*^**						0.074
**Low-grade**	979 (54.1)	173 (55.4)	438 (51.4)	350 (57.3)	18 (52.9)	
**High-grade**	830 (45.9)	139 (44.6)	414 (48.6)	261 (42.7)	16 (47.1)	
**Missing**	50	11	18	19	2	
***Tumor number***						0.256
**Single**	1,030 (59.7)	179 (60.9)	466 (57.6)	364 (61.8)	21 (65.6)	
**Multiple**	694 (40.3)	115 (39.1)	343 (42.4)	225 (38.2)	11 (34.4)	
**Missing**	135	29	61	41	4	
***Tumor size***						0.379
**< 3cm**	377 (75.1)	60 (73.2)	171 (72.8)	139 (78.5)	7 (87.5)	
**≥ 3cm**	125 (24.9)	22 (26.8)	64 (27.2)	38 (21.5)	1 (12.5)	
**Missing**	1,357	241	635	453	28	
***Histological type***						0.126
**UCC**	1,808(97.8)	310 (96.9)	848 (98.1)	616 (98.1)	34 (94.4)	
**SCC**	17 (0.9)	5 (1.6)	7 (0.8)	5 (0.8)	0 (-)	
**AC**	9 (0.5)	4 (1.2)	1 (0.1)	3 (0.5)	1 (2.8)	
**Other**	14 (0.8)	1 (0.3)	8 (0.9)	4 (0.6)	1 (2.8)	
**Missing**	11	3	6	2	0	

^1^ P value is based on chi-square, Fisher exact, or one-way ANOVA test, where appropriate.

^2^ Tumors with WHO 1973 differentiation grade 1 or 2, WHO/ISUP 2004 low grade, or Malmström (Modified Bergkvist) grade 1 or 2a were considered low-grade tumors. Tumors with WHO 1973 differentiation grade 3, WHO/ISUP 2004 high grade, or Malmström (Modified Bergkvist) grade 2b or 3 as high-grade

Missing data were not included in the calculation of p values. Abbreviations: N: number of patients; SD: standard deviation; CIS: carcinoma *in situ*; UCC: urothelial cell carcinoma; SCC: Squamous cell carcinoma; AC: Adenocarcinoma.

Table 3 shows the distribution of smoking variables in different tumor aggressiveness groups and different tumor multiplicity groups, by smoking group. In former cigarette smokers, there is a significant difference in the smoking amount, smoking duration and cumulative smoking in the subgroups of aggressiveness. The median of smoking amount is higher in the high-risk NMIBC compared with low-aggressive NMIBC, but lower in MIBC. In former smokers, smoking duration is highest in patients with MIBC and lowest in patients with low risk NMIBC. The median time since smoking cessation was highest in patients with low-risk NMIBC and lowest in patients with MIBC. In ever and current cigarette smokers, there was no difference in the distribution of smoking variables between the subgroups of aggressiveness. There doesn’t seem to be a strong correlation between smoking and tumor multiplicity. If anything, only current smokers may have more frequently solitary tumors.

**Table 3:** Distribution of cigarette smoking habits according to tumor aggressiveness and tumor multiplicity at first diagnosis.

	All urinary bladder cancer (n = 1,859)^1^(Median (Q_3_-Q_1_);_ _N missing)	Low-risk NMIBC(n = 867) (Median (Q_3_-Q_1_))	High- risk NMIBC (n = 646)(Median (Q_3_-Q_1)_)	MIBC (n = 322)(Median (Q_3_-Q_1_))	*p* value^2^	Solitary (n = 1,009)(Median (Q_3_-Q_1_))	Multiple (n = 683)(Median (Q_3_-Q_1)_)	*p* value^2^
***Smoking status***					<0.001^3^			0.256^4^
**Never cigarette smokers (%)**	323 (17.7)	154 (18.1)	115 (18.1)	49 (15.7)		179 (17.7)	115 (16.8)	
**Ever cigarette smokers (%)**	1500 (82.3)	698 (81.9)	519 (81.9)	264 (84.3)		830 (82.3)	568 (83.2)	
**Smoking amount (cig/day)**	15 (20–10);8	15 (20–10)	15 (20–10)	15 (20–10)	0.142	15 (20–10)	15 (20–10)	0.314
**Smoking duration (y)**	32 (42–20);85	32 (42–20)	31 (40–20)	35 (43–23)	0.055	33 (42–20)	31 (41.8–21)	0.683
**Cumulative smoking (pack-years)**	22.5 (35–12);91	22.4 (34.5–10.5)	23 (35.9–13)	22.5 (36–12.5)	0.363	22.5 (36–12)	23 (34–12.6)	0.583
**Age at initiation (y)**	16 (18–15):3	16 (18–15)	16 (18–15)	16 (18–15)	0.870	16 (18–15)	17 (18–15)	0.121
**Former cigarette smokers (%)**	870 (47.7)	386 (45.3)	340 (53.6)	134 (42.8)		466 (46.2)	343 (50.2)	
**Smoking amount (cig/day)**	15 (20–10); 7	14 (20–10)	15 (20–10)	11.5 (20–8)	0.008	15 (20–10)	15 (20–10)	0.259
**Smoking duration (y)**	26 (37–17); 39	25 (35–15)	27 (37–18.3)	29.5 (40–19)	0.007	25 (36.3–16.8)	26 (37–18)	0.381
**Cumulative smoking (pack-years)**	18 (30–8.8); 44	16 (26.3–7.4)	20 (31.8–10.2)	18.375 (29.8–8.4)	0.003	16 (27.8–7.5)	18.9 (30–9.6)	0.112
**Age at initiation (y)**	17 (18–15); 2	17 (18–15)	16 (18–15)	17 (19–15)	0.231	17 (18–15)	17 (18–15)	0.566
**Time since smoking cessation (y)^5^**	18 (26–10); 0	19 (28–11)	17 (24.75–10)	15.5 (24–9)	0.034	17 (26–9)	18 (26–11)	0.354
**Current cigarette smokers (%)**	630 (34.6)	312 (36.6)	179 (28.2)	130 (41.5)		364 (36.1)	225 (32.9)	
**Smoking amount (cig/day)**	15 (20–10); 1	15 (20–10)	15 (20–10)	15 (20–10)	0.783	15 (20–10)	15 (20–10)	0.002
**Smoking duration (y)^6^**	40 (47–30); 46	40 (48–30)	40 (47–30)	39.5 (47–31)	0.859	40 (47–32)	39 (48–30)	0.701
**Cumulative smoking (pack-years)^6^**	28.5 (40.8–19); 47	29 (41.4–19.4)	27 (40.5–18.4)	27 (39.9–18.1)	0.728	30.6 (41.3–20)	26 (39–16.3)	0.024
**Age at initiation (y)**	16 (18–15);1	16 (18–15)	16 (18–15)	16 (18–15)	0.173	16 (18–15)	16 (18–15)	0.117

^1^ Missing tumor aggressiveness in 24 patients, missing tumor multiplicity in 135 patients and missing smoking status in 36 patients.

^2^ P value is based on non-parametric Kruskal-Wallis one-way ANOVA test.

^3^ The distribution of smoking status is significantly different between the three tumor aggressiveness groups (based on chi-square test)

^4^The distribution of smoking status is not significantly different between the two tumor multiplicity groups (based on chi-square test)

^5^ Time elapsed since smoking cessation was calculated as the difference between the age at diagnosis and reported age at cessation.

^6^ Corrected for number of smoking years after diagnosis.

Abbreviations: NMIBC: non-muscle-invasive bladder cancer; MIBC: muscle-invasive bladder cancer; Q_1_: first quartile; Q_3_: third quartile; cig/day: cigarettes per day; y: years.

In multinomial regression analyses with adjustment for age at diagnosis and gender (Table 4), smoking amount was a risk factor of getting high-risk NMIBC compared with low-risk NMIBC in ever and former cigarette smokers (OR: 1.02 per cigarette smoked a day, 95% CI: 1.00–1.03 and OR: 1.03, 95% CI: 1.01–1.05, respectively). Smoking duration was a risk factor for MIBC compared with low-risk NMIBC in ever and former cigarette smokers (OR per year smoked: 1.01, 95% CI: 1.00–1.03 and OR: 1.02, 95% CI: 1.00–1.04, respectively). Time since smoking cessation was a protective factor in both comparisons, i.e., a longer time of smoking cessation leads to a lower odds of a more aggressive cancer. Smoking intensity variables were not significantly related to tumor aggressiveness in current cigarette smokers. By contrast, only in current smokers there seems to be an association between smoking duration and tumor multiplicity suggesting that a longer smoking history leads to a higher risk of solitary tumors.

**Table 4:** Multivariable regression analyses of smoking intensity in relation to tumor aggressiveness and tumor multiplicity.

	High- risk NMIBC vs. low- risk NMIBC (OR (95% CI))^1^	*p* value	MIBC vs. low- risk NMIBC (OR (95% CI))^1^	*p* value	Multiple vs. Solitary (OR (95% CI))^1^	*p* value
***Smoking status***						
**Ever cigarette smokers**						
**Smoking amount (cig/day)**	1.02 (1.00–1.03)	0.019	1.00 (0.99–1.02)	0.871	1.00 (0.98–1.01)	0.541
**Smoking duration (y)^3^**	1.00 (1.00–1.01)	0.487	1.01 (1.00–1.02)	0.036	1.00 (0.99–1.00)	0.229
**Cumulative smoking (pack-years)^3^**	1.00 (1.00–1.01)	0.306	1.01 (1.00–1.01)	0.271	1.00 (0.99–1.00)	0.302
**Age at initiation (y)**	1.00 (0.97–1.03)	0.732	0.99 (0.95–1.03)	0.535	1.02 (0.99–1.05)	0.134
**Former cigarette smokers**						
**Smoking amount (cig/day)**	1.03 (1.01–1.05)	0.002	1.01 (0.98–1.03)	0.588	1.01 (0.99–1.02)	0.317
**Smoking duration (y)**	1.01 (1.00–1.03)	0.083	1.02 (1.00–1.04)	0.027	1.00 (0.99–1.01)	0.795
**Cumulative smoking (pack-years)**	1.01 (1.01–1.02)	0.003	1.01 (1.00–1.02)	0.134	1.00 (1.00–1.01)	0.416
**Age at initiation (y)**	0.97 (0.92–1.02)	0.185	0.96 (0.91–1.02)	0.223	1.01 (0.97–1.05)	0.677
**Time since smoking cessation (y)^2^**	0.98 (0.97–0.99)	0.006	0.97 (0.96–0.99)	0.006	1.00 (0.99–1.02)	0.682
**Current cigarette smokers**						
**Smoking amount (cig/day)**	0.99 (0.97–1.02)	0.525	0.99 (0.96–1.02)	0.646	0.96 (0.94–0.99)	0.005
**Smoking duration (y)^3^**	0.99 (0.97–1.01)	0.435	1.00 (0.98–1.03)	0.873	0.99 (0.97–1.01)	0.140
**Cumulative smoking (pack-years)^3^**	1.00 (0.98–1.01)	0.506	1.00 (0.98–1.01)	0.710	0.99 (0.98–1.00)	0.015
**Age at initiation (y)**	1.02 (0.98–1.06)	0.425	1.00 (0.96–1.05)	0.895	1.03 (1.00–1.07)	0.085

^1^ Adjusted for age at diagnosis (continuous) and gender.

^2^ Time elapsed since smoking cessation was calculated as the difference between the age at diagnosis and reported age at cessation.

^3^ Corrected for number of smoking years after diagnosis.

Abbreviations: NMIBC: non-muscle-invasive bladder cancer; MIBC: muscle-invasive bladder cancer; cig/day: cigarettes per day; y: years.

Table 5 presents the results from multinomial logistic regression after including smoking duration as a categorical instead of continuous variable. A longer smoking duration is associated with higher risks of MIBC compared with low-risk NMIBC mostly in ever and former cigarette smokers (p value for trend: 0.004 and 0.008, respectively). Comparing high-risk NMIBC with low-risk NMIBC, there is a higher odds ratio with increasing smoking duration until 30 years. Thereafter, the risk remains the same or even decreases. In former smokers, there is a significantly increasing trend (p value: 0.035).

**Table 5:** Multivariable regression analyses of smoking duration in relation to tumor aggressiveness.

	N (%)	High-risk NMIBC vs. Low-risk NMIBC (OR (95% CI))^1^	*p* value	MIBC vs. Low-risk NMIBC (OR (95% CI))^1^	*p* value
***Smoking status***					
**Ever cigarette smokers**					
**Smoking duration^2^^,^^3^**			0.871		0.004
**<10 years (ref)**	84 (5.9)	1	-	1	-
**10-<20 years**	211 (14.9)	1.47 (0.82–2.63)	0.192	1.55 (0.70–3.40)	0.280
**20-<30 years**	299 (21.1)	1.72 (0.98–3.00)	0.057	1.71 (0.80–3.66)	0.166
**30-<40 years**	359 (25.4)	1.73 (1.00–2.99)	0.051	2.10 (1.00–4.41)	0.050
**≥40 years**	462 (30.8)	1.32 (0.76–2.28)	0.328	2.42 (1.16–5.06)	0.018
**Former cigarette smokers**					
**Smoking duration^3^**			0.035		0.008
**<10 years (ref)**	74 (8.9)	1	-	1	-
**10-<20 years**	174 (20.9)	1.54 (0.83–2.84)	0.172	1.68 (0.70–4.07)	0.248
**20-<30 years**	223 (26.8)	1.81 (0.99–3.31)	0.054	1.90 (0.79–4.52)	0.150
**30-<40 years**	197 (23.7)	2.05 (1.11–3.81)	0.023	1.92 (0.79–4.70)	0.152
**≥40 years**	163 (19.6)	2.00 (1.03–3.86)	0.039	3.29 (1.33–8.10)	0.010
**Current cigarette smokers**					
**Smoking duration^2^^,^^3^**			0.920		0.353
**<10 years (ref)**	10 (1.6)	1	-	1	-
**10-<20 years**	37 (6.3)	1.47 (0.25–8.72)	0.673	1.11 (0.18–6.88)	0.912
**20-<30 years**	76 (13.0)	2.16 (0.40–11.68)	0.373	1.05 (0.19–5.85)	0.960
**30-<40 years**	162 (27.7)	1.95 (0.38–10.15)	0.428	1.57 (0.30–8.23)	0.597
**≥40 years**	299 (51.2)	1.48 (0.28–7.82)	0.645	1.51 (0.28–8.25)	0.631

^1^ Adjusted for age at diagnosis (continuous) and gender.

^2^ Corrected for number of smoking years after diagnosis.

^3^ *p* value for trend

NMIBC: non-muscle-invasive bladder cancer; MIBC: muscle-invasive bladder cancer; y: years.

## Discussion

The present study was performed with the goal of examining the association between smoking intensity and tumor aggressiveness. Different aspects of smoking intensity were evaluated such as smoking amount in cigarettes per day, smoking duration and cumulative smoking in pack-years. Significant but weak positive associations were found in ever cigarette smokers concerning smoking amount and smoking duration. When the ever cigarette smokers were separated in former and current cigarette smokers, inconsistent results were found. In the subgroup of former cigarette smokers, the same relations were found as in ever cigarette smokers, but in the subgroup of current cigarette smokers no clear relation was found. Apparently, the results in ever smokers were driven by those in former smokers only. In the analyses of tumor multiplicity the reverse was seen. Only in current smokers, there was a significantly lower risk of multiple tumors with a longer smoking history.

Among ever smokers, smoking amount was a significant risk factor for high-risk NMIBC compared with low-risk NMIBC, but not for MIBC compared with low-risk NMIBC. The same holds true with smoking duration among ever and former smokers and cumulative smoking in former smokers. These conflicting results suggest that the relation between smoking intensity and tumor aggressiveness is probably weak or even just a chance finding. Previous studies [20–28] showed no dose-response relations between tumor characteristics and smoking intensity, supporting our findings.

Jiang et al. [15] combined tumor grade and stage to classify tumor aggressiveness and found that the risk of more advanced tumors was positively associated with smoking duration and smoking intensity. Unfortunately, the authors did not make a distinction between former and current cigarette smokers like we did.

In our study, a longer time of smoking cessation was associated with a lower risk of an aggressive bladder cancer. In contrast, Jiang et al. [15] showed that an increasing number of years since quitting leads to a decreased risk of UBC, but there was no difference among subgroups of tumor aggressiveness.

*FGFR3* mutations are associated with low-grade and low-stage tumors while *TP53* mutations are associated with high-grade and high-stage tumors [2,34]. If smoking leads to different mutations in these or other stage-related genes, then it would be logical to find an association between smoking and disease aggressiveness. It has been shown in lung cancer, based on data from The Cancer Genome Atlas (TCGA), that smoking induces specific gene mutations [35]. However, this does not seem to be the case in bladder cancer. In The Cancer Genome Atlas there was no statistically significant association between smoking status and the mutational spectrum, frequency of mutation in any significantly mutated gene, occurrence of focal somatic CNAs or expression subtype in 131 muscle invasive bladder cancers (although it is not clear from the paper how ‘smoking’ was phenotyped). However, a major subtype of MIBC (‘CIMP’) was identified by unsupervised cluster analysis that has high-level promoter hypermethylation associated with the number of pack-years smoking [36]. In a follow-up paper describing a larger number of tumors (N = 409) somewhat more *ERCC2* signature mutations are found among smokers [37]. A small study from Canada showed that *TP53* mutations are more common with increasing years of smoking, although not more common with increasing numbers of cigarettes smoked [38].

McConkey et al. [39] and other groups have recently suggested a new molecular subclassification of bladder cancer related with distinct patterns of progression and response to conventional chemotherapy. It is possible that this new classification is stronger related to the intensity of smoking. Indeed, in a recent study, again based on TCGA, it was shown that patients with the more aggressive basal-like subtypes, started smoking earlier than patients with a luminal subtype [40]. Unfortunately, we were unable to examine this in our study.

A strength of the present paper is its large sample size. A weakness is that the NBCS had a retrospective design which means that prevalent cases were recruited for the study. Especially patients with more severe disease may have deceased prior to recruitment. The time lag between diagnosis and study enrollment was up to 12 years. The absence of prevalent patients that failed to survive until the sampling date has resulted in a study population biased towards favorable tumor aggressiveness. In theory, this may have biased the effect size estimates and thereby the ability of our study to identify any relation between smoking and tumor aggressiveness. For that reason, we repeated our analyses using only the subset of our patient cohort with a maximum time between diagnosis and study enrollment of 3 years (approximately 50% of the series). This analysis showed only marginal differences with the results using the whole series.

If there is a shorter diagnostic delay among smokers, it might cause a bias towards an association between smoking and a more favorable disease stage. In The Netherlands, there is no screening or active case finding for bladder cancer. In the case of unexplained macroscopic hematuria, the guidelines dictate that bladder cancer should be ruled out. We believe that the adherence to this guideline is very good in men, irrespective of their smoking status. It is generally known that the diagnostic delay is somewhat longer in women. We cannot rule out that smoking habits may have influenced this diagnostic delay in some women.

In theory, our study may have suffered from differential misclassification if patients with a more aggressive tumor reported their smoking habits in a different way than patients with a less aggressive tumor. Unfortunately, there is no way to check this. Of course, there will have been a certain degree of non-differential misclassification of smoking habits. Also, we did not collect information on, e.g., the use of filter cigarettes, depth of inhaling, brand of the cigarette, and passive smoking. It is impossible to capture all of these differences but a prospective design with serial measurements may lead to less misclassification compared to the single retrospective measurement in our study.

For a large percentage of patients, tumor size was missing due to a lack of information in the medical records. This prohibited us to evaluate an association between smoking intensity and this characteristic.

Among former cigarette smokers, larger smoking amount and longer smoking duration are weakly related with a more aggressive cancer but no relation was found among current smokers.

This inconsistency may suggest that there is no strong relation between smoking intensity and aggressiveness of the tumor. However, the retrospective design of the study may have influenced the results to some extent. Analyses of prospective studies with longitudinal smoking assessment might answer the research question in a more definitive way.
